# ANTHROPOMETRIC CORRELATION OF HAMSTRING AND PERONEUS LONGUS GRAFTS IN ACL RECONSTRUCTION

**DOI:** 10.1590/1413-785220263404e300989

**Published:** 2026-07-24

**Authors:** Izabela Feres de Oliveira, Matheus Kuster Ronconi, Gabriel Velasque dos Santos Midão, Fabrício Nascimento de Almeida, Gustavo Dalla Bernardina de Almeida, Saulo Gomes de Oliveira

**Affiliations:** 1 Hospital Santa Casa de Misericordia de Vitoria, Vitoria, ES, Brazil.

**Keywords:** Anthropometry, Anterior Cruciate Ligament Injuries, Transplants, Tendons, Antropometria, Lesões do Ligamento Cruzado Anterior, Transplantes, Tendões

## Abstract

This study aimed to analyze the association between anthropometric data of patients with anterior cruciate ligament injuries and the characteristics of the grafts used in ligament reconstruction. This analytical study involved 30 patients randomly assigned to two groups: Group I, flexor tendon grafts (semitendinosus and gracilis), and Group II, peroneus longus tendon grafts. Graft length and diameter were measured, as well as preoperative anthropometric variables such as age, height, and weight. Student's t-test, chi-square test, and Pearson's correlation test were used. The results showed significantly greater length for the flexor tendon grafts (28.8 ± 2.66 vs. 25.8 ± 3.19 cm), while the peroneus longus tendon exhibited a greater final diameter (9.33 ± 0.82 vs. 8.47 ± 0.52 cm), both with p < 0.05. A negative correlation was observed between age and the length of the peroneus longus graft, and a positive correlation between height and the same parameter. Anthropometric data were not shown to be reliable predictors for the flexor grafts, although younger and taller patients were more likely to have a greater length in the peroneus longus graft. **Level of Evidence I; Prognostic study – investigation of the effect of a patient's characteristics on disease outcome.**

## RESUMO

O estudo objetivou analisar a associação entre os dados antropométricos de pacientes com lesão do ligamento cruzado anterior e as características dos enxertos empregados na reconstrução ligamentar. Trata-se de um estudo analítico com 30 pacientes, distribuídos aleatoriamente em dois grupos: Grupo I, enxerto dos tendões flexores (semitendíneo e grácil), e Grupo II, enxerto do tendão fibular longo. Foram mensurados o comprimento e o diâmetro dos enxertos, além de variáveis antropométricas no pré-operatório, como idade, estatura e peso. Utilizou os testes t de Student, Qui-quadrado e Correlação de Pearson. Os resultados mostraram comprimento significativamente maior para os enxertos dos tendões flexores (28,8 ± 2,66 vs. 25,8 ± 3,19 cm,), enquanto o tendão fibular longo exibiram diâmetro final superior (9,33 ± 0,82 vs 8,47 ± 0,52 cm), ambos com p < 0,05. Observou-se correlação negativa entre idade e comprimento do enxerto fibular longo, e correlação positiva entre estatura e o mesmo parâmetro. Os dados antropométricos não se mostraram preditores confiáveis para os enxertos flexores, embora pacientes mais jovens e mais altos tenham maior probabilidade de comprimento superior no enxerto fibular longo. **Nível de Evidência I; Estudo prognóstico – investigação do efeito de característica de um paciente sob o desfecho da doença.**

## INTRODUCTION

The anterior cruciate ligament (ACL) originates from the posterior face of the lateral femoral condyle and inserts laterally and anteriorly to the medial tibial spine. The ACL has an average intra-articular length of 38 mm and an average diameter of 11 mm,^1^ with its primary function being the anterior stabilization of the tibia and restriction of internal rotation of the knee.^2^

ACL rupture is the most common ligament injury of the knee.^3^ Among the recommended treatments, surgical method is the primary choice, with intra-articular reconstruction via arthroscopy being the most common.^4^ According to the Ministry of Health in Brazil, the incidence of ACL reconstruction procedures is 3.49 cases per 100,000 people per year, highlighting the importance of this treatment in the Brazilian population within the Unified Health System (SUS).^5^

During ACL reconstruction, the choice of the appropriate graft is crucial for the success of the procedure. Autologous grafts, such as those from the quadriceps tendons, patellar tendon, flexors (semitendinosus and gracilis), and peroneus longus, are commonly used due to their availability and biocompatibility.^6^ Studies show that grafts with diameters less than 7 mm present a higher risk of failure and recurrence,^7^ emphasizing the importance of adequate size to ensure postoperative durability. Each type of graft has its particularities regarding length, diameter, and presents specific advantages and disadvantages concerning postoperative functional impairment, such as muscle recovery of the donor area, postoperative pain, and risk of long-term complications.^8^

It is known that the long fibular tendon graft has high resistance, safety, and lower morbidity; however, few studies demonstrate its surgical disadvantages, unlike the hamstrings, which are widely studied, already showing loss of strength and a long incorporation time (ligamentization).^9^

In light of the above, this study aims to compare the autologous grafts of the flexors (semitendinosus + gracilis) (STG) to that of the long fibular (FL), correlating these data with the anthropometric characteristics of the patients, collected preoperatively. In order to analyze whether there is a statistical difference between the characteristics of the grafts, Student's *t* tests (simple and paired) and Chi-Square tests were used to evaluate differences between groups and associations. Additionally, correlations between anthropometric data and graft characteristics were analyzed using Pearson correlation, whose values range from -1 to 1, indicating a direct or inverse relationship.

## MATERIALS AND METHODS

This is an observational, cross-sectional, analytical correlation study involving patients with complete ACL rupture, scheduled for RACL reconstruction at the knee outpatient clinic of a philanthropic hospital, between June and December 2024. The research was initiated after approval from the Ethics Committee with CAAE number 82144124.2.0000.5065.

Thirty patients were included, randomly divided by the Research Randomizer site into two groups of 15 individuals, according to the type of autologous tendon harvested for RACL reconstruction, being: Group I, flexor graft (semitendinosus + gracilis tendons); and Group II, long fibular tendon.

The collection of anthropometric information was conducted through a questionnaire during the preoperative consultation, after they agreed and signed the Informed Consent Form (ICF).

The patient's weight was measured using a digital scale with a precision of one decimal place. Height was measured with a non-elastic tape measure with the patient in an orthostatic position with bipodal support.^10^ Other anthropometric data were obtained through a questionnaire.

**Figure 1:**
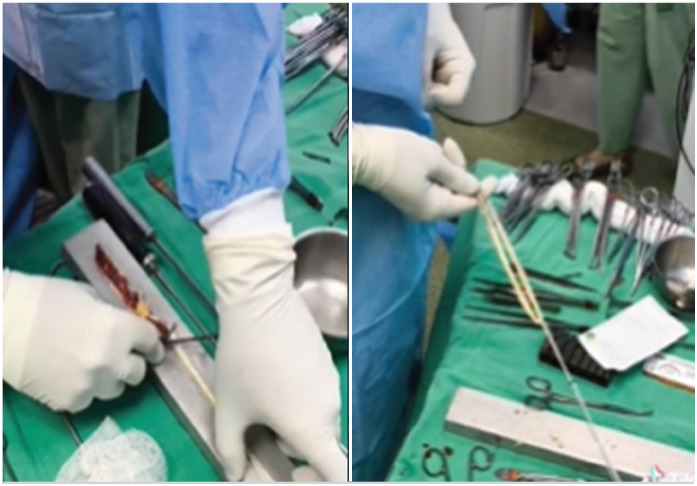
Preparation of the collected graft.

**Figure 2:**
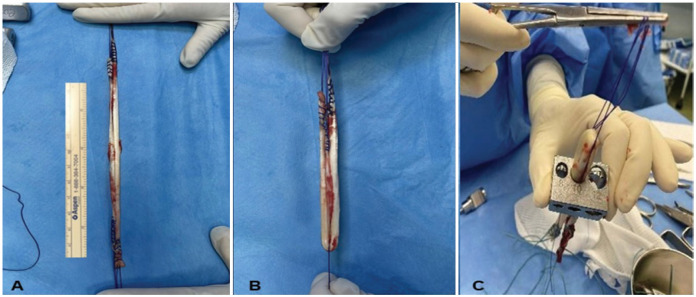
A- Measurement of the graft length after preparation, using calibrated surgical instruments. B- Graft folded after measuring the length. C- Measurement of the graft diameter with a standardized gauge.

In Group I, the semitendinosus and gracilis tendons were harvested through a 2–3 cm incision on the anteromedial face of the knee, at the insertion of the Pes Anserinus, about 4–5 cm below the medial joint line,^11^ using Stripper instruments. Subsequently, they were subjected to the removal of muscle fragments and prepared on the surgical table with the aid of forceps and high-strength sutures (Ethibond No. 2), where their longitudinal edge was sutured to keep the edges of the semitendinosus and gracilis tendons together, using Krakow suture (Figure 1). After this preparation, their length was measured with a sterile millimeter ruler, obtaining the final length of the graft. Finally, the graft was folded according to the plan, achieving a minimum final length of 9 cm, with its diameter measured using a standardized gauge (Figure 2).

**Figure 3:**
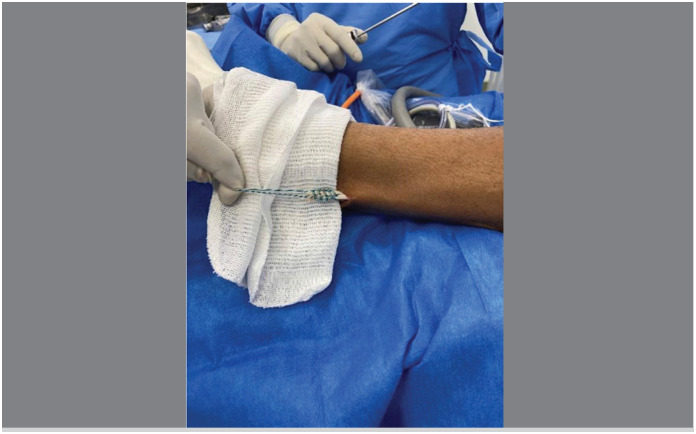
Joining of the distal stump of the long fibular tendon graft with the intact short fibular tendon.

In Group II, the graft was harvested through an incision on the lateral face of the ankle, posterior to the lateral malleolus, with the aid of the Stripper. The distal portion was preserved and sutured to the short fibular tendon (tenodesis) to maintain part of the muscular function of the long fibular (Figure 3).^12^ The same measurements were taken in Group I after preparation.

The data obtained from the anthropometric measurements and grafts were transferred to a table in the software Microsoft Office/ Excel 2011 (Redmond, Washington, USA).

The statistical methodology used Student's t-tests (simple and paired) to assess differences between groups, and the Chi-Square test to identify associations. The correlations between anthropometric data and graft characteristics were analyzed using Pearson's correlation, whose values range from -1 to 1, indicating a direct or inverse relationship.

A confidence interval for the mean and p-value was used, with a significance level of 0.05. The data from the grafts and anthropometric correlations were analyzed using parametric or non-parametric tests, depending on the distribution. The statistical analysis was performed using the software SPSS Statistics 28.0.1. P values < 0.05 indicated statistical significance.^13^

## RESULTS

The Table 1 shows a comparison of the anthropometric factors of Groups I (Flexor) and II (Fibular) regarding height, age, and weight, where the values ranged from 166-195 cm, 17-52 years, and 68-97 kg (Flexor), and 153-185 cm, 22-66 years, and 60-117 kg (Fibular). In general, the results demonstrated an absence of statistical difference, indicating homogeneity between the groups.

**Table 1:** Comparison of the Anthropometric Factors of Groups I (Flexor) and II (Fibular).

		Mean	Median	Standard Deviation	CV	Min	Max	N	IC	P-value
Age	Flexor	30.3	25	12.5	41%	17	52	15	6.3	0.189
	Fibular	37.1	38	14.8	40%	22	66	15	7.5	
Height	Flexor	176.0	175	7.4	4%	166	195	15	3.7	0.065
	Fibular	170.3	170	8.7	5%	153	185	15	4.4	
Weight	Flexor	80.5	77	9.1	11%	68	97	15	4.6	0.354
	Fibular	85.0	80	16.3	19%	60	117	15	8.2	

**Figure 4:**
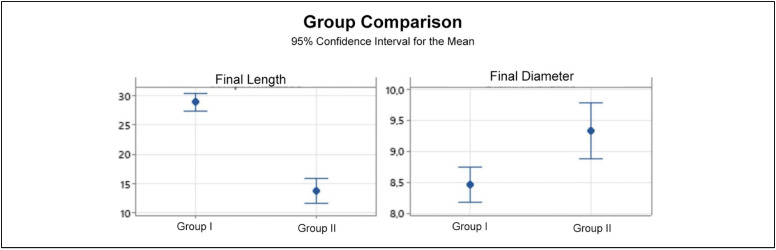
Comparison of length (cm) and diameter (cm) of the finished grafts by group (I and II).

The comparative analysis of the mean lengths of the grafts showed a statistically significant difference, with a greater mean length in Group I (Flexors), 28.80 cm (±2.66), compared to Group II (Long Fibular), 25.80 cm (±3.19), with p=0.009. Additionally, there was a difference in the mean diameters, greater in Group II, Long Fibular graft, 9.33 mm (±0.82) compared to Group I, Flexor graft, 8.47 (±0.52) with (p 0.002) (Table 2 and Figure 4)

**Table 2:** Comparison of groups I and II of the average length and diameter of the completed grafts.

		Average	Median	Standard Deviation	CV	Min	Max	N	IC	P-value
Length of the Completed Graft	Group I	28.80	28.5	2.66	9%	24	33	15	1.31	0.009
	Group II	25.80	26	3.19	12%	20	31	15	1.61	
Diameter of the Completed Graft	Group I	8.47	8	0.52	6%	8	9	15	0.26	0.002
	Group II	9.33	9	0.82	9%	8	11	15	0.41	

**Figure 5:**
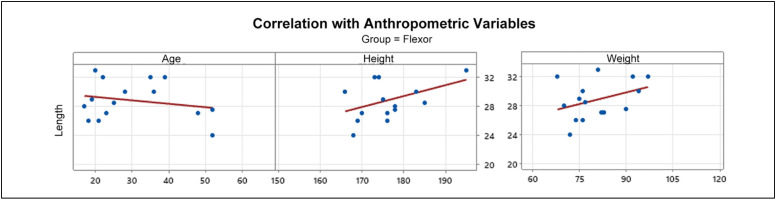
Correlation between the anthropometric measurements of age, height, and weight with the parameter of the finalized graft length, Group I, Flexor Tendon.

**Figure 6:**
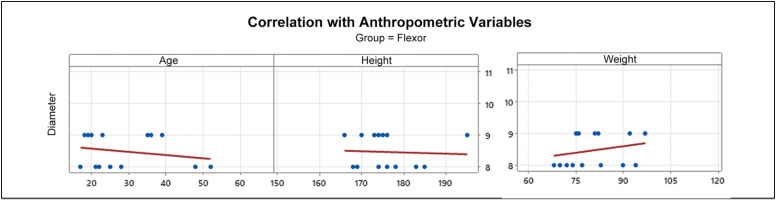
Correlation between the anthropometric measurements of age, height, and weight with the parameter of the finalized graft diameter, Group I, Flexor Tendon.

**Figure 7:**
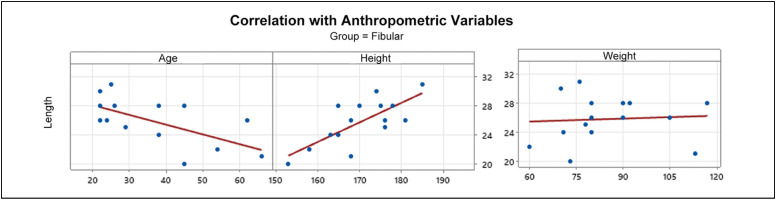
Correlation between the anthropometric measurements of age, height, and weight with the parameter of the finalized graft length, Group II, Long Fibular Tendon.

**Figure 8:**
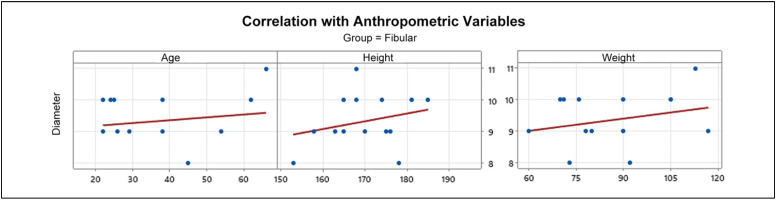
Correlation between the anthropometric measurements of age, height, and weight with the parameter of the finalized graft diameter, Group II, Long Fibular Tendon.

The correlation between anthropometric measurements (age, height, and weight) with the length and diameter of the grafts was analyzed in both groups (Table 3). Age showed a negative correlation with the length of the Long Fibular graft, (r= -0.622), with (p=0.013), indicating an inversely proportional variation, where a lower age corresponds to a greater length of the obtained long fibular graft. Height showed a positive correlation with the length of the Long Fibular graft, (r= 0.736), with (p=0.002), indicating a directly proportional variation, where a greater height of the patient corresponds to a greater length of the obtained long fibular graft. These variables did not show a statistically significant correlation with the flexor tendon graft, nor did weight in either of the two groups. (Figures 5-8).

**Table 3:** Correlation of anthropometric factors with tendon measurements by group.

		Age		Height		Weight	
		Corr (r)	P-value	Corr (r)	P-value	Corr (r)	P-value
Group I	Completed Length	-0.248	0.372	0.404	0.135	0.354	0.196
	Completed Diameter	-0.247	0.375	-0.056	0.843	0.240	0.389
Group II	Length of the Completed Graft	-0.518	0.048	0.475	0.073	-0.047	0.868
	Diameter of the Completed Graft	0.164	0.560	0.265	0.341	0.258	0.353

## DISCUSSION

When comparing our findings with the literature, we found an average length of the flexor tendons after preparation, before being folded, of (28.80 ± 2.66 cm), which was considerably higher than the flexor graft reported in other studies. Wan et al^14^ found average lengths of flexor grafts close to 25 cm.

Regarding the length of the Long Fibular graft, Khan et al.,^15^ report an average length of 321.4 ± 26.7 mm, a value higher than that found in our study (25.80 ± 3.19 cm), however, a sufficient length, as stated by Janssen et al.,^16^ who indicated minimum lengths of 21 cm sufficient for complex anatomical ligament reconstructions of the ACL.

Goyal et al.,^17^ highlighted the minimum graft length for ACL reconstruction, finalized after the fold, of 8 cm in length. Our case series achieved a graft length that allows for folds in both groups, without compromising the useful length, ensuring adequate filling of the bone tunnels and stability of the fixation.

Similarly, the length of the found flexor tendons allows for use in combined or multiligament reconstructions, where several distinct tunnels and greater tissue demand are necessary, especially in the group of Flexors that were statistically larger than the Long Fibular tendon, whose use as a graft in these surgeries requires caution due to the lack of adequate length in some situations.^18^

The long fibular graft had a statistically greater diameter than the flexor in our case series: greater in relation to Group I (Flexors): 8.47±0.52 vs. Group II, (Long Fibular): 9.33 mm±0.82 (p= 0.002). Gupta et al. (2018), evaluating 156 patients undergoing ACL reconstruction with long fibular tendon graft, found an average diameter of 8.3 mm, having a significant correlation with height, weight, and injury duration, allowing for the development of a predictive equation for graft caliber.^19^ Our results demonstrated an average diameter of the Long Fibular graft superior to that reported by Gupta et al., which may be justified by distinct anthropometric and ethnic factors in each sample.

We also observed a significant negative correlation between the length of the long fibular graft and the age of the patients (r = –0.518; p = 0.048), indicating that younger individuals tend to have larger caliber grafts. Thus, we reinforce the likely biomechanical advantage of the long fibular as a safe and effective graft, demonstrating it to be an excellent graft option in this group of patients, given that grafts ≥ 8 mm present a lower risk of failure.^18^ However, more clinical and biomechanical studies need to confirm this thesis, reinforcing whether there is superiority in ACL reconstructions with Long Fibular graft in younger patients, given the larger average diameter in this population.

Another finding demonstrates a positive correlation between height and final length of the long fibular graft, being directly proportional to the greater height of the patient with the longer fibular graft. We did not find data or studies evaluating these characteristics in the medical literature, possibly due to the recent interest in the long fibular graft and few published works on the subject.

Thus, considering that there was a statistical correlation between the anthropometric data and the measurements of the grafts, and that the grafts of the flexor tendons are longer and those of the long fibular tendon have a greater diameter, it is possible to guide surgical planning according to the need: opting for the flexors when greater length is required, and for the long fibular when there is a need for a graft with greater diameter, with possible advantages of stiffness and resistance.

## CONCLUSION

The results of this study demonstrated that the grafts of the flexor tendons have significantly greater length, while the long fibular tendon has a superior final diameter, both with statistical significance. It was also observed that age presented a negative correlation and height a positive correlation with the length of the long fibular graft, while the other anthropometric variables did not show reliable predictors. These findings reinforce the importance of considering the specific characteristics of each graft in the choice of the reconstructive technique for the anterior cruciate ligament, indicating that the long fibular tendon may represent a valid alternative when seeking greater diameter, especially in younger and taller patients.

## Data Availability

The underlying content of the research text is contained in the manuscript.
